# The Effect of Micrococcal Nuclease Digestion on Nucleosome Positioning Data

**DOI:** 10.1371/journal.pone.0015754

**Published:** 2010-12-29

**Authors:** Ho-Ryun Chung, Ilona Dunkel, Franziska Heise, Christian Linke, Sylvia Krobitsch, Ann E. Ehrenhofer-Murray, Silke R. Sperling, Martin Vingron

**Affiliations:** 1 Department of Computational Molecular Biology, MPI für Molekulare Genetik, Berlin, Germany; 2 Group Cardiovascular Genetics, Department of Vertebrate Genomics, MPI für Molekulare Genetik, Berlin, Germany; 3 Abteilung für Genetik, ZMB, Universität Duisburg-Essen, Essen, Germany; 4 Neurodegenerative Disorder Group, Otto Warburg Laboratory, MPI für Molekulare Genetik, Berlin, Germany; National Institute on Aging, National Institutes of Health, United States of America

## Abstract

Eukaryotic genomes are packed into chromatin, whose basic repeating unit is the nucleosome. Nucleosome positioning is a widely researched area. A common experimental procedure to determine nucleosome positions involves the use of micrococcal nuclease (MNase). Here, we show that the cutting preference of MNase in combination with size selection generates a sequence-dependent bias in the resulting fragments. This strongly affects nucleosome positioning data and especially sequence-dependent models for nucleosome positioning. As a consequence we see a need to re-evaluate whether the DNA sequence is a major determinant of nucleosome positioning *in vivo*. More generally, our results show that data generated after MNase digestion of chromatin requires a matched control experiment in order to determine nucleosome positions.

## Introduction

The genomes of eukaryotic organisms are packaged into chromatin, whose basic repeating unit is the nucleosome [1]. The nucleosome forms by the association of two copies each of the four core histones, H2A, H2B, H3 and H4 forming the histone octamer and ∼147 base pairs of DNA. The DNA adopts a flat left handed superhelix with ∼1.65 turns around the histone octamer [2]. All processes acting with and on the DNA are taking place in a chromatinized environment. For example, the histone octamer competes with transcription factors for access to the DNA, suggesting that the positioning of nucleosomes relative to *cis*-regulatory sequences plays a major role in transcriptional regulation. Thus, “Unraveling the rules and factors that determine how nucleosomes are positioned and how they influence gene activity and evolution is one of the central questions in biology today.” [3]

Key to answer these questions is the identification of nucleosome positions on a genome wide scale. A common experimental approach to identify nucleosome positions involves the use of Micrococcal nuclease (MNase). MNase preferentially cuts linker DNA connecting two nucleosomes, while the nucleosomal DNA is at least partially protected against MNase digestion [4], [5]. Given these properties, MNase has been used to map nucleosomes on a small scale by the indirect labeling approach [6], [7]. However, MNase cuts DNA in a sequence-dependent manner [8], [9], suggesting that the cutting frequency can vary even in the absence of a nucleosome. To control for this feature of MNase subsequent studies included a control digestion of naked DNA [10], [11], [12], [13]. In recent genome-wide studies on nucleosome positioning the paradigm shifted from the identification of nucleosomal DNA fragments directly by means of a reduced cutting frequency to a more indirect one that measured the abundance of recovered nucleosome sized DNA fragments by microarrays [14], [15], [16], [17], [18], [19] or by deep sequencing [20], [21],[22],[23],[24],[25],[26],[27],[28].

These studies provide rich datasets to unravel the determinants of nucleosome positioning (reviewed in [3], [29]). For example, it has been found that nucleosome positions depend on the action of ATP-dependent remodeling complexes [18], the binding of transcription factors [20], [30], RNA-polymerase [27], [31]. Moreover, it has been found that budding yeast nucleosome occupancies measured *in vivo* by deep sequencing are accurately predictable using the genomic sequence only, indicating that the DNA sequence plays a major role in positioning nucleosomes *in vivo* [23].

Recently, the important role of the DNA sequence in nucleosome positioning has been called into question. It has been found that fission yeast utilizes different sequence-dependent positioning rules than budding yeast [15], a finding which is not compatible with the notion of a general (eukaryotic) nucleosome positioning code. Moreover, it has been argued that the number of recovered DNA fragments is not necessarily proportional to the nucleosome occupancy due to systematic errors introduced by the MNase digestion and/or the deep sequencing [32], [33]. Here, we will assess the impact of MNase digestion on the number of DNA fragments by measuring the abundance of DNA fragments recovered after MNase digestion of naked DNA and size selection of ∼150 base pair fragments. We will show that the resulting coverage profile is very similar to the one obtained by digesting chromatin, suggesting that MNase digestion and the size selection indeed leads to a systematic measurements bias, which explains both the high correlation between *in vitro* and *in vivo* nucleosome occupancy profiles and the good predictive power of the computational model.

## Results and Discussion

**Figure 1:**
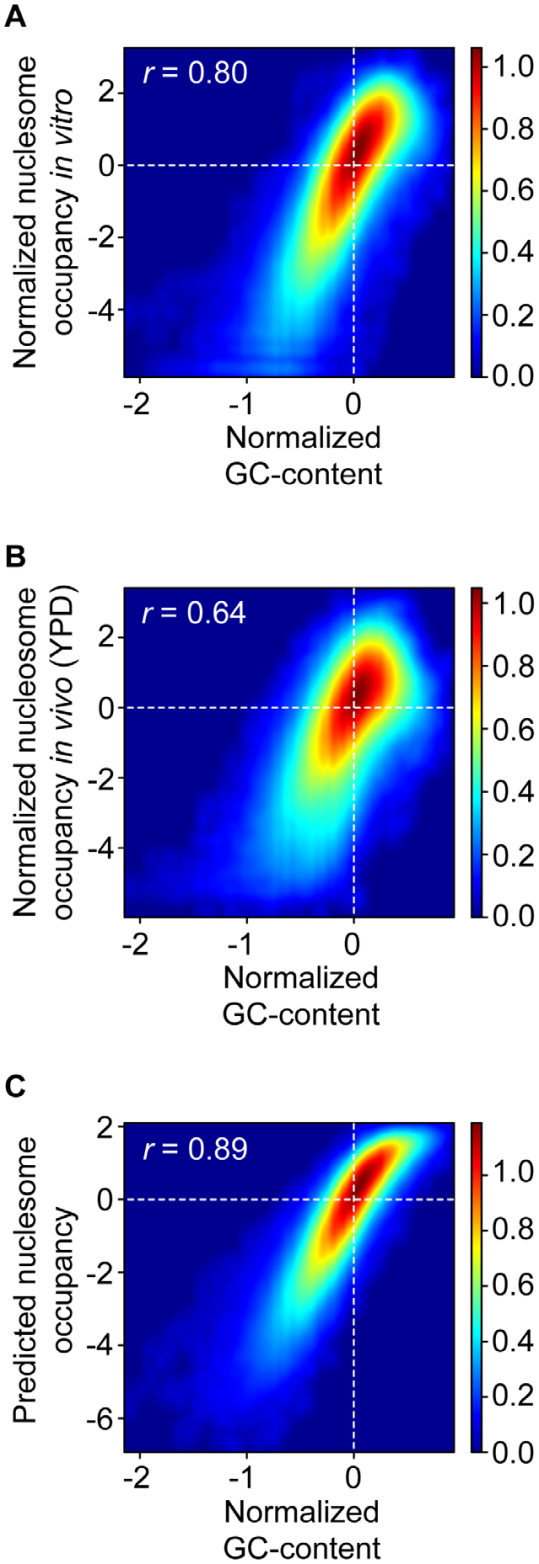
The GC content in 147 base pair windows is strongly correlated to nucleosome occupancy. Density plot comparison between the normalized centered GC content in 147 base pair windows (*x* axis) and (**A**) the *in vitro* reconstituted, (**B**) the *in vivo* (YPD) and (**C**) the predicted nucleosome map (*y* axis). The color of each point represents the density of base pairs mapping to it, where the density-color relationship is shown at the right of each plot. The Pearson correlation coefficients *r* between the data sets is indicated.

Nucleosomal DNA sequences tend to be enriched in GC base pairs, implying that GC-richness promotes nucleosome formation [34], [35], [36]. Accordingly, the log_2_ transformed and normalized GC-content within 147 base pairs correlates with the *in vitro* reconstituted (Pearson correlation *r* = 0.80), *in vivo* (*r* = 0.64), and predicted nucleosome occupancies (*r* = 0.89; Figure 1), implying that the GC content is a major nucleosome positioning determinant and contributes significantly to the prediction. However, MNase cuts almost exclusively at AT base pairs [8], [9] and is able to cut nucleosomal DNA [4], [37]. We hypothesized that the size selection step prior to end-sequencing might enrich for GC-rich DNA fragments, because those have a lower probability of internal cuts (and vice versa AT-rich DNA fragments might be depleted due to their higher probability of internal cuts). To test whether the use of MNase combined with the size-selection biases the measured nucleosome positions, we digested naked yeast DNA with MNase, size-selected ∼150 bp fragments and deep sequenced one of the two ends of the recovered fragments (see Materials and Methods for details).

**Figure 2:**
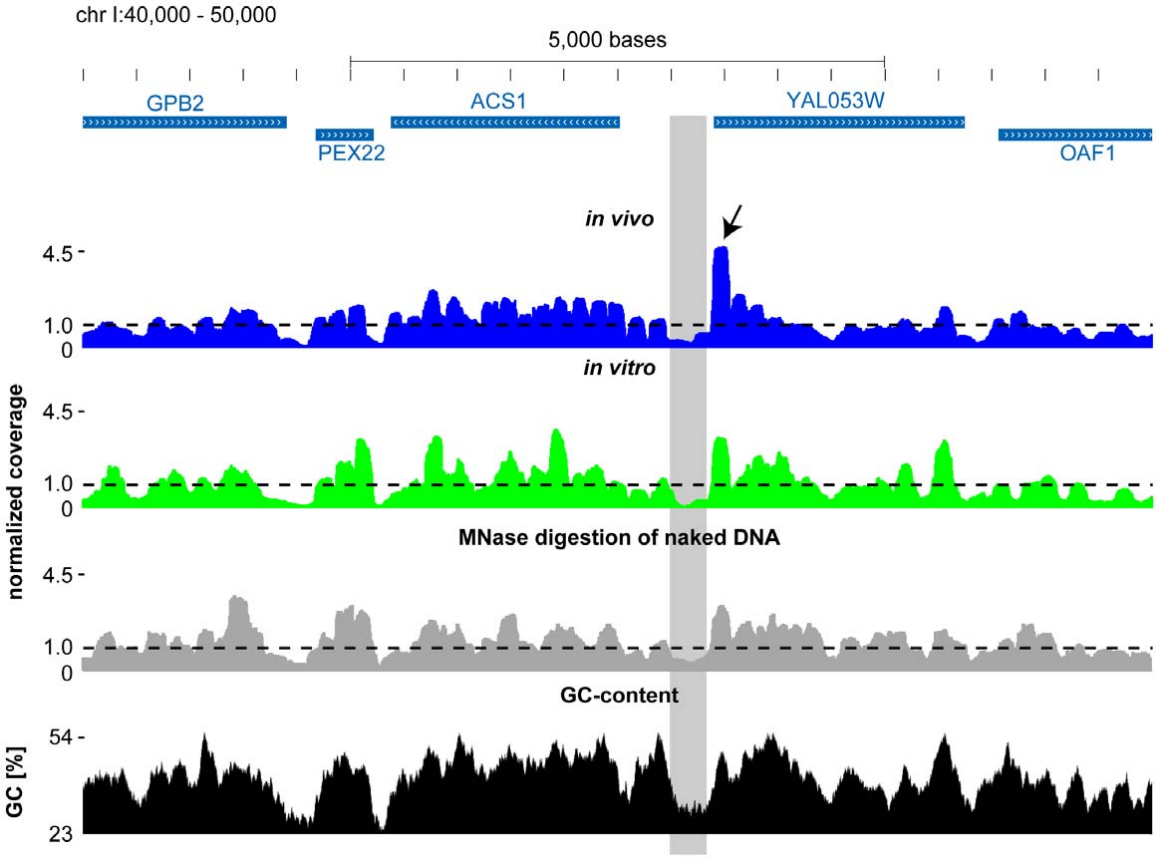
Exemplary profiles in a region of chromosome I. Shown is an exemplary region (40,000 to 50,000 base pairs) of chromosome I of *S. cerevisiae*. The blue boxes mark the open reading frames of annotated genes, where the direction of the arrowheads correspond to the direction of transcription. The profiles of the *in vivo* (blue), *in vitro* (green) and the MNase digestion of naked DNA (gray) are shown underneath. The y axes of these profiles correspond to the nucleosome occupancy/coverage per base pair normalized by dividing by the genome wide average. The dashed line at 1.0 therefore corresponds therefore to the genome wide average. The last track shows the GC-content (black) in percent in 147 base pair windows centered around the central base pair. The arrow indicates the presumable +1 nucleosome of YAL053W and the gray box marks the nucleosome depleted region immediately upstream (see Text for details).

An exemplary region is shown in Figure 2. The three profiles are similar but not identical. It can be seen that whenever the coverage in the naked DNA digestion profile is very low (or very high), the corresponding *in vitro* and *in vivo* profiles are very low (or very high) as well. For example, the *in vivo* occupancy profile (shown in blue) has a very pronounced peak in the vicinity of the start codon of YAL053W (marked with an arrow in Figure 2), which presumably corresponds to the +1 nucleosome immediately downstream of the transcriptional start site of this gene. A similar peak can be seen in the *in vitro* (shown in green) as well as the naked DNA digestion profile (shown in gray), suggesting that the high occupancy is at least in part due to systematic measurement biases. In line with the idea that the experimental procedure leads to an enrichment of GC-rich sequences, there is also a peak in the GC-content (shown in black) centered on the +1 nucleosome of YAL053W. Upstream of the +1 nucleosome, there is a nucleosome depleted region in the *in vivo* and *in vitro* profile (marked with a gray box in Figure 2). The same region also shows low coverage in the naked DNA digestion profile accompanied with AT-richness (or GC depletion), suggesting that the apparent nucleosome depletion measured both *in vivo* an *in vitro* can at least in part be explained by the sequence preferences of MNase in conjunction with the size selection step.

**Figure 3:**
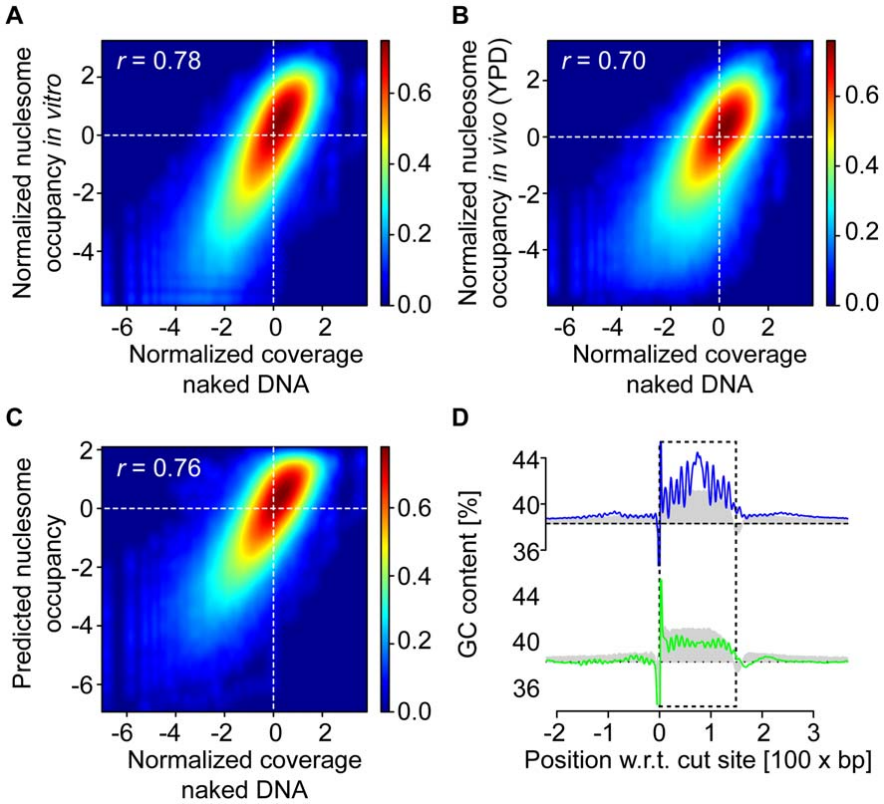
MNase digestion leads to systematic measurement bias. (**A**–**C**) Density plot comparison between the normalized coverage per base pair in the MNase generated map on naked DNA (*x* axis) and (**A**) the *in vitro* reconstituted, (**B**) the *in vivo* (YPD) and (**C**) the predicted nucleosome map (*y* axis). The color of each point represents the density of base pairs mapping to it, where the density-color relationship is shown at the right of each plot. The Pearson correlation coefficients *r* between the data sets are indicated. (**D**) The GC-content profile in percent (3 base pair moving average) around the start coordinate of the reads. The gray filled curve corresponds to the profile obtained by digesting naked DNA with MNase and the blue and green curve represent the profile for the *in vitro* and *in vivo* (YPD) data, respectively. The dashed horizontal line indicates the average GC content in the yeast genome (38.3%) and the box indicates the selected fragment (0–149 base pairs).

In order to get a systematic measure of similarity genome wide, we calculated the Pearson correlation coefficient *r* between the different profiles (after taking the binary logarithm). This analysis shows that the resulting log_2_ transformed and normalized coverage per base pair is strongly correlated to the *in vitro* reconstituted (*r* = 0.78; Figure 3A), *in vivo* (YPD, *r* = 0.70; Figure 3B) and predicted nucleosome occupancies (*r* = 0.76; Figure 3C). The Pearson correlation coefficients are very similar to the ones obtained by comparing the *in vitro* to *in vivo* (*r* = 0.74), the predicted to *in vitro* (*r* = 0.89) and predicted to *in vivo* data (*r* = 0.75) [23].

In line with our hypothesis that MNase digestion coupled to size selection leads to an enrichment of GC-rich sequences, we found that genome wide the GC-content of the DNA fragments recovered after MNase digestion of naked DNA exhibit an increase of GC-content in the fragments like the *in vitro* and *in vivo* experiments. However, these lack the 10 base pair oscillatory signal in the GC-content present in both the *in vitro* and *in vivo* data (Figure 3D), suggesting that the former is MNase-, while the latter is nucleosome-specific. In support for this idea, there is a much weaker but very well visible 10 base pair oscillatory signal just upstream of the sequenced and size-selected DNA fragments *in vitro* and *in vivo*, which is not accompanied by an increase in GC-content.

**Figure 4:**
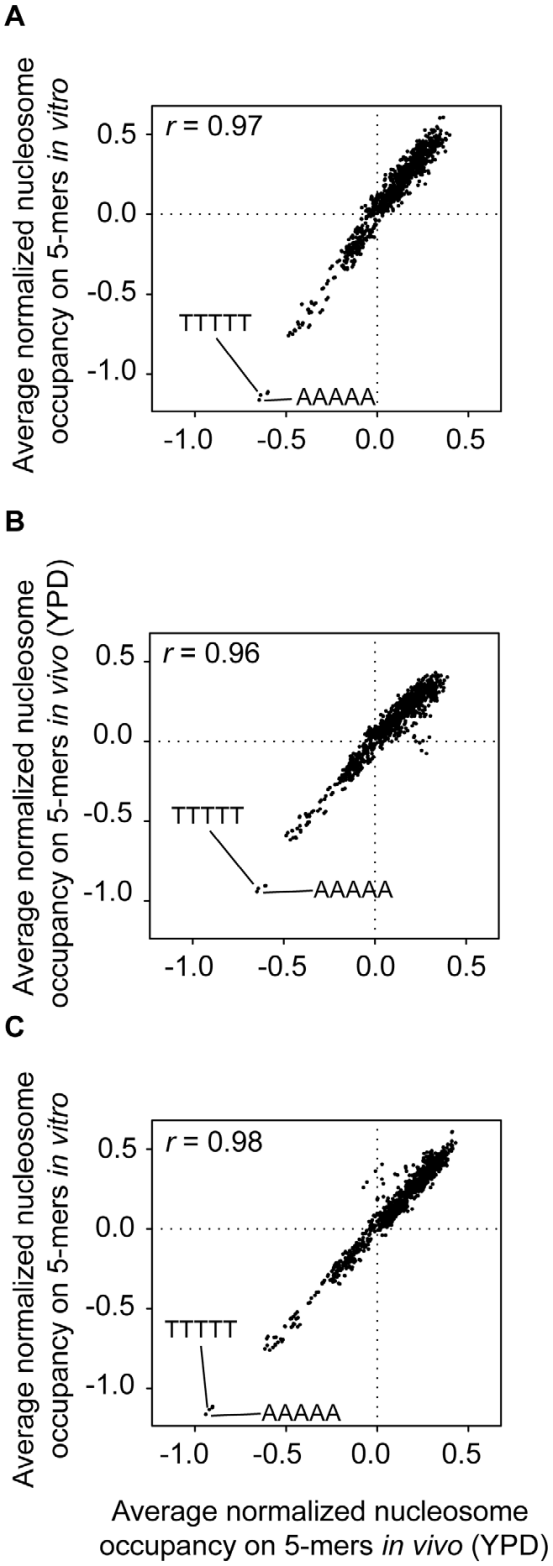
Comparison of the average genome wide relative coverage of sequence of length 5. (**A**) the MNase generated map on naked DNA (*x* axis) and the *in vitro* reconstituted nucleosome map (*y axis*); (**B**) the MNase generated map on naked DNA (*x* axis) and the *in vivo* (YPD) nucleosome map (*y axis*); (**C**) the *in vivo* (YPD) nucleosome map (*x axis*) and the *in vitro* nucleosome map (*y axis*). The Pearson correlation coefficients *r* between the data sets are indicated.

Furthermore, we found that when looking at the genome-wide relative nucleosome occupancies over sequences of length 5, our data almost perfectly recapitulates both the *in vitro* (*r* = 0.97) and the *in vivo* (*r* = 0.96) data (Fig. 4). In particular, the 5mers AAAAA/TTTTT, proposed to be a nucleosome exclusion signal [38], have the lowest average coverage in all three data sets.

Our results, suggest that MNase digestion together with size selection leads to an enrichment of GC-rich and a depletion of AT-rich sequences. In line with this interpretation of our result it was found that sequence signatures associated with nucleosomes are also recovered upon considering control data in which genomic DNA was either sonicated or digested with MNase in the absence of nucleosomes (see http://arxiv.org/abs/1003.4044). Another study revealed that the depletion of nucleosomes at the 3′ termini of genes seen in MNase dependent experiments cannot be reproduced by a MNase-independent approach [39]. 3′ termini of genes are AT-rich, suggesting that the depletion of nucleosomes seen in MNase dependent experiments are likely to be the consequence of the sequence preferences of MNase.

In the worst case scenario some GC-rich DNA fragments can be recovered even without protection by the histone octamer. In order to check whether this is a possibility we focused on the Gal1-10 locus. For this locus, nucleosome positions have been determined by cutting chromatin with bleomycin [40]. Using this method it has been found that the upstream activating sequence (UAS) common to the divergently transcribed Gal1 and Gal10 genes is nucleosome depleted when culturing yeast in rich medium containing 2% glucose. This nucleosome depleted region is flanked by a two highly positioned nucleosomes and the degree of positioning decreases with increasing distance to the UAS, consistent with a statistical nucleosome positioning scenario [41]. Moreover, it has been shown that this UAS is DNAse I [42], [43] and (methiumpropyl−EDTA)iron(II) sensitive [44]. Finally, it has been shown that this region is bound by GAL4 and GAL80 [45].

**Figure 5:**
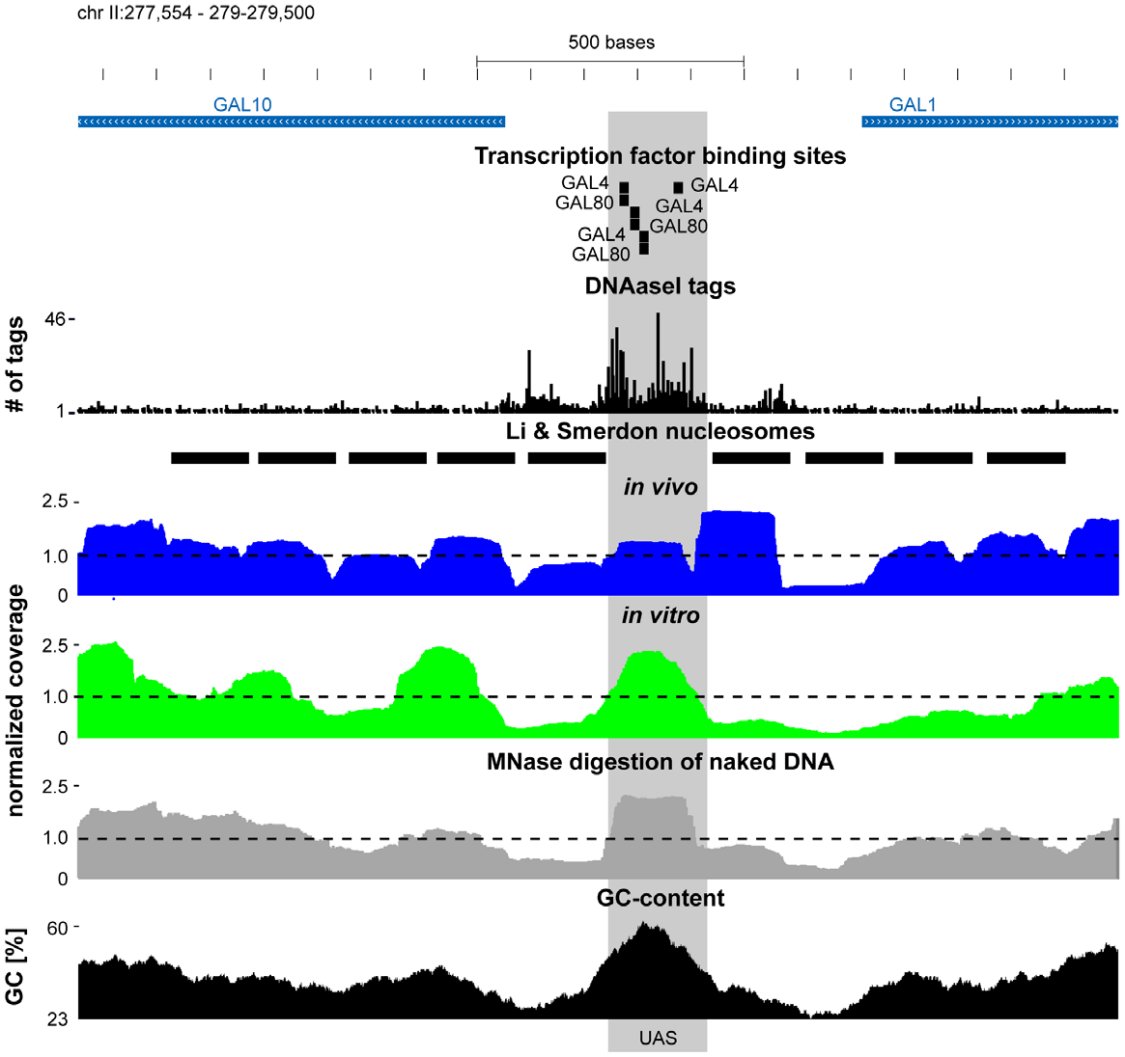
Nucleosome positioning in the Gal1-10 Locus. Shown is the Gal1-10 locus on chromosome II (277,554 to 279500). The blue boxes mark the open reading frames of annotated genes, where the direction of the arrowheads correspond to the direction of transcription. The transcription factor binding sites are from Harbison *et al.* (2004) [45]. The DNAse I tag numbers are from Hesselberth *et al.* (2009) [42], shown on the y axis is the number of tags for each position. Nucleosome positions determined by Li and Smerdon (2002) are denoted as black boxes [40]. The profiles of the *in vivo* (blue), *in vitro* (green) and the MNase digestion of naked DNA (gray) are shown underneath. The y axes of these profiles correspond to the nucleosome occupancy/coverage per base pair normalized by dividing by the genome wide average. The dashed line at 1.0 therefore corresponds therefore to the genome wide average. The last track shows the GC-content (black) in percent in 147 base pair windows centered around the central base pair. The gray box denotes the UAS of Gal1 and Gal10.

A comparison of the occupancy/coverage profiles is shown in Figure 5. The nucleosome occupancy profile measured in YPD *in vivo* (shown in blue) shows no marked depletion at the UAS, but reveals even a well positioned nucleosome covering the UAS. Thus, the nucleosome depletion at the UAS cannot be reproduced by the MNase digestion, size selection and deep sequencing approach. The reason for this becomes evident by looking at the other profiles. The *in vitro* profile (shown in green) shows a peak at the UAS region, which is also present in the naked DNA profile (shown in gray), suggesting that the nucleosome signal covering the UAS *in vivo* might originate from the MNase bias. In line with the idea that MNase digestion together with size selection leads to an enrichment of GC-rich sequences, the UAS is indeed very GC rich (see Figure 5 shown in black). Thus, it seems that a well established nucleosome depleted region, the UAS of Gal1 and Gal10, cannot be reproduced by MNase digestion, size selection and deep sequencing due to the systematic sequence-dependent measurement biases.

Our results indicate that the increase of GC-content in the recovered fragments can be attributed to the sequence preference of MNase in conjunction with the size selection step, explaining to some degree the correspondence between the *in vitro*, *in vivo* and predicted nucleosome occupancy maps. However, our results cannot explain that the predicted nucleosome occupancy, which is largely dependent on the GC content, correlates also with nucleosome occupancies derived by MNase independent approaches [23], [46]. These studies support the claim that the histone octamer prefers GC-rich sequences. If true, MNase might just prefer sequences which are disfavored by the histone octamer, explaining the high correlation between the profiles obtained by digesting *in vitro* reconstituted chromatin and naked DNA. However, the nucleosome occupancies measured by these MNase independent approach have been derived from material that has been reconstituted *in vitro* by a method called salt-gradient dialysis. This method has been shown to select sequences that are well bound by the H3/H4 tetramer but not necessarily by the full octamer. In fact, it has been shown that the H2A/H2B dimers contribute only very little to the total binding energy [47]. In line with this finding it has been observed that the H3/H4 tetramer binds DNA at very high salt concentrations, while the H2A/H2B dimers bind at lower salt concentrations [48]. Moreover, it has been shown that the H3/H4 tetramer prefers GC-rich sequences in high salt conditions [48]. Given that DNA is a polyelectrolyte, its physical properties, like bendability and curvature, will depend on the salt concentration. Thus, at this point it is unclear whether the GC preference of the H3/H4 tetramer at high salt concentrations is also present at physiological salt conditions. Finally, it has been shown that the full histone octamer minimally prefers AT-rich sequences [49]. In light of these results it seems likely that nucleosomes assembled by salt gradient dialysis form at sequences that are GC-rich due to the preference of GC-rich sequences by the H3/H4 tetramer at high salt concentrations. Thus, the correlation between predicted and MNase independently measured nucleosome occupancy may be due to two unrelated biases which both enrich for GC-rich sequences.

In an earlier study, we have proposed that the GC-content is one of the major sequence-dependent determinant of nucleosome positioning [34]. In this study, we analyzed nucleosome positioning data obtained by MNase digestion, chromatin immunoprecipitation, size selection and sequencing. Thus, the GC-content increase in the recovered DNA fragments observed also in this dataset is likely to be due to the sequence preferences of MNase in combination with size selection.

Taken together, we showed that the nucleosome positioning data generated by MNase digestion might reflect some aspects of the real nucleosomal landscape. But in the absence of a suitable control experiment, i.e. similar MNase activity to DNA ratio and similar size-selection, the measured nucleosome positions are biased by the experimental procedures. Thus, we conclude that the MNase generated nucleosome positioning data alone is insufficient to determine nucleosome positions and holds only limited evidence to claim that the DNA sequence is a major determinant of nucleosome positioning.

## Materials and Methods

### Datasets

The normalized occupancies per base pair were downloaded from http://genie.weizmann.ac.il/pubs/nucleosomes08/nucleosomes08_data.html and the predicted occupancies from http://genie.weizmann.ac.il/software/nucleo_genomes.html. The genome sequence was downloaded from http://genie.weizmann.ac.il/software/data/S0106.fa.gz. This is the genome version used in [23] and should ensures comparability to the data reported in [23] The raw reads for the *in vitro* reconstituted nucleosomes were downloaded from the Short Read Archive at NCBI using the accession numbers SRR023798 and SRR023799. The raw reads for the *in vivo* (YPD) nucleosomes were downloaded from NCBI using the accession numbers SRR023800 to SRR023805.

### Deep sequencing of MNase treated naked yeast genomic DNA

We followed closely the experimental procedures described in [23] for the parallel sequencing of nucleosomes reconstituted *in vitro* on yeast genomic DNA, with some modifications. Briefly, *S. cerevisiae* genomic DNA was isolated from strain BY4741 (*MATa his3Δ1 leu2Δ0 met15Δ0 ura3Δ0*) using standard methods and RNA was removed by an additional RNase A treatment for 30 min at 37°C. The pellet was resuspended in TE and passed 10 times through a 25 gauge needle and then 10 times through a 27 gauge needle. The DNA was run on a 22×16 cm, 0.8% agarose TAE gel for 7h at 100V at 4°C. The genomic DNA band was excised from the gel and the DNA was eluded by electrophoresis in a dialysis bag (Spectra/Por Dialysis Membran MWCO: 3,500 18mm, Spectrum Laboratories, Inc). The DNA was precipitated and the pellet was resuspended in 10mM Tris pH 8.0, 1mM CaCl_2_. The DNA was digested with 6×10^−3^ units micrococcal nuclease (Sigma Chemical Company) per 10 µg genomic DNA at 37°C for 5 min. The reaction was stopped by the addition of 1 µl 0.5M EDTA. The DNA was precipitated and after resuspension in 10mM Tris pH 8.0, 1mM CaCl_2_ electrophoresed on a 6% polyacrylamide TBE gel for 30 min at 200 V and a band corresponding to 140 to 170 base pairs was excised. The DNA was eluded. Library preparation and end-sequencing was performed by the NGS service facility at the Max-Planck-Institut für molekulare Genetik.

### Mapping of the sequencing reads

The reads were mapped to the yeast genome using RazorS [50]. We also mapped the raw reads for the *in vitro* reconstituted as well as the *in vivo* nucleosomes to determine the GC content profile. For the micrococcal nuclease digested naked DNA 10,988,458 reads out of the 18,717,648 reads mapped to the yeast genome. For each genomic coordinate the number of reads mapping to the + strand and the − strand were determined.

### Determination of the average fragment length

A histogram of the distances between every + strand read and − strand read in a window of 300 base pairs was generated and the average fragment size was determined by summing the distances weighted by their abundance.

### Normalized coverage map

The normalized coverage map was generated by extending each + strand read to by +150 base pairs and each − strand read by −150 base pairs and summing the values for each base pair in the yeast genome. Thereafter, the counts were transformed by taking the binary logarithm. The values were normalized by setting the genome wide average to zero by subtracting the mean transformed counts.

### Normalized GC content in a window 147 base pairs

The GC content was determined in windows of 147 base pairs and assigning the value to the central base pair, i.e. the value found at position 74 corresponds to the GC content of a sequences starting at position 1 and ending at position 147. The values were transformed by taking the binary logarithm. The values were normalized by setting the genome average to zero by subtracting the mean transformed GC content.

### Density plots

For the density plots, we extracted the values for each data set that were common to all data sets. In total there were 10,727,725 value pairs.

### GC content profiles

The GC content profiles were computed by aligning all reads at their start coordinate (the − strand reads were reverse complemented) and counting the occurrences of the bases at each position starting from −2000 and ending at +2000 base pairs. The resulting GC content profile had a strong 3 base pair periodic component (due to the codons). Therefore, a 3 base pair moving average was applied on the GC frequencies.

### Average normalized coverage over sequences of length 5

The average normalized coverage over sequences of length 5 was computed by averaging the mean coverage value over the corresponding 5 base pairs, only sequences of length 5 were considered that had values for each of the 5 base pairs.
